# A Facile Method to Realize Oxygen Reduction at the Hydrogen Evolution Cathode of an Electrolytic Cell for Energy-Efficient Electrooxidation

**DOI:** 10.3390/ma14112841

**Published:** 2021-05-26

**Authors:** Zhiqiang Zhao, Lu Liu, Luofu Min, Wen Zhang, Yuxin Wang

**Affiliations:** State Key Laboratory of Chemical Engineering, Tianjin Key Laboratory of Membrane Science and Desalination Technology, School of Chemical Engineering and Technology, Tianjin University, Tianjin 300072, China; zqzhao@tju.edu.cn (Z.Z.); 1016207153@tju.edu.cn (L.L.); minluofu@tju.edu.cn (L.M.); zhang_wen@tju.edu.cn (W.Z.)

**Keywords:** carbon black, gas electrode, ORR, in situ anodization, APS, electricity saving

## Abstract

Electrochemical oxidation, widely used in green production and pollution abatement, is often accompanied by the hydrogen evolution reaction (HER), which results in a high consumption of electricity and is a potential explosion hazard. To solve this problem, we report here a method for converting the original HER cathode into one that enables the oxygen reduction reaction (ORR) without having to build new electrolysis cells or be concerned about electrolyte leakage from the O_2_ gas electrode. The viability of this method is demonstrated using the electrolytic production of ammonium persulfate (APS) as an example. The original carbon black electrode for the HER is converted to an ORR electrode by first undergoing in situ anodization and then contacting O_2_ or air bubbled from the bottom of the electrode. With this sole change, APS production can achieve an electric energy saving of up to 20.3%. Considering the ease and low cost of this modification, such significant electricity savings make this method very promising in the upgrade of electrochemical oxidation processes, with wide potential applications.

## 1. Introduction

Electrochemical oxidation is an important part of electrolysis and electrosynthesis, both of which use electrons as reagents and control the rate and direction of reactions by adjusting the electrode potential, thus constituting an effective means of green chemistry [1,2,3,4,5,6]. Electrochemical oxidation, realized through direct electron transfer and/or by reducing the oxidants generated in situ, can be used to produce a variety of inorganic and organic chemicals, including chlorine gas [7], potassium permanganate [8], ammonium persulfate [9], ozone [10], benzaldehyde [11], trifluoroacetic acid [12], p-anisaldehyde [11], etc. It is also widely used in wastewater treatment, especially in the degradation of refractory organic pollutants in water [13,14,15,16].

However, many current electrochemical oxidation processes are accompanied by the hydrogen evolution reaction (HER) at the counter electrode. The accompanying HER not only causes a high energy consumption but can also lead to fire and explosion hazards as a result of the released hydrogen gas mixing with the surrounding air [9,17,18,19,20]. To protect against hazards, facilities for collecting and safely diluting or burning the released hydrogen are usually needed, resulting in complicated operational processes and additional investment and operation costs. Alternatively, the HER can be replaced by the electrochemical oxygen reduction reaction (ORR). The ORR proceeds at much higher potential than the HER does and produces water, meaning that the electrical energy used to drive an electrochemical oxidation process can be saved and hazards related to hydrogen release can be avoided [21]. At present, researchers have conducted a significant amount of work on the ORR and have accumulated experience for the preparation of an efficient oxygen reducing catalyst [22,23,24,25,26].

As oxygen presents a very limited solubility in aqueous solutions—e.g., only around 8 ppm in 25 °C water—the ORR on a common electrode immersed in electrolyte would be quite slow [26]. Conventionally, an ORR that is industrially viable is realized via a gas diffusion electrode (GDE), which enables the direct supply of reactive gases to the “three-phase interface” where the electrochemical reaction takes place. A GDE has a complicated porous structure and needs to achieve a proper hydrophobic–hydrophilic balance so as to ensure an unobstructed gas supply while preventing any leakage of the electrolyte. A successful example of using a GDE to realize the ORR is in chlor-alkali electrolysis, where up to 30% of electricity is saved with the so-called “oxygen depolarized cathode” (ODC) [27,28]. However, because of the complexity and frequently higher cost, ORR on a GDE has not found wide application.

In this paper, we propose a simple method to realize a practical ORR without employing a conventional GDE. Our gas electrode is composed of carbon black (CB) supported on a titanium mesh and bound with polytetrafluoroethylene (PTFE). The carbon black is anodized in situ to increase its ORR activity. The gas electrode is used by immersing it in the electrolyte and supplying it with oxygen gas via bubbles generated beneath it. This arrangement completely avoids any possible leakage of electrolyte, meaning that issues seen in examples using a GDE, such as pore structure and hydrophobic–hydrophilic balance, do not have to be addressed. The feasibility and effect of the proposed method are demonstrated using the electrosynthesis of ammonium persulfate (APS) as an example. APS was chosen because it is produced in large quantities and dominantly synthesized via electrolysis [29,30]. In addition, APS electrosynthesis is known for its high energy consumption [31,32].

## 2. Experimental

### 2.1. Reagents and Materials

PTFE emulsion (60 wt %) was obtained from Aladdin Reagent (Shanghai, China) Co., Ltd. Nafion115 cation exchange membrane was from Shanghai Hesen Electronic Device Co., Ltd. Carbon black (Vulcan XC-72) was from Shanghai Macklin Biochemical Co., Ltd. (Shanghai, China) Titanium mesh (40 mesh) was bought from Hongyun Metal Products Co., Ltd. (Shanghai, China). Oxygen (4N) was from Tianjin Dongxiang Speciality Gas Co., Ltd. (Tianjin China) Ammonium polyphosphate (*n* < 20) was obtained from Shanghai Xinbo Chemical Technology Co., Ltd. (Shanghai, China) NaOH, H_2_SO_4_, (NH_4_)_2_SO_4_, FeSO_4_·7H_2_O, H_3_PO_4_, K_2_Cr_2_O_7_, and absolute ethanol were purchased from Tianjin Kemiou Chemical Reagent Co., Ltd. (Tianjin, China). All the reagents in the experiment were used without further separation and purification.

### 2.2. Preparation and Activation of the CB Electrode

The CB electrode was prepared using PTFE-bound CB supported on titanium mesh. The received Ti mesh was treated in 0.14 mol·L^−1^ of NaOH solution at 90 °C for 30 min, followed by rinsing with deionized water and drying at 120 °C before use. Carbon paste was prepared by first mixing 0.6 g of carbon black and 0.5 g of 60 wt % PTFE emulsion in 10 mL of absolute ethanol, then evaporating solvents in the mixture at 70 °C while stirring until the mixture became very thick. The carbon paste was spread on Ti mesh and hot-pressed under 10 MPa at 100 °C for 15 min. This was followed by sintering at 300 °C for 10 min to obtain the CB electrode. The electrode thus obtained can be used directly for a HER but requires modification for use in an ORR.

To achieve better ORR performance, the CB electrode was electrochemically modified via anodization. An oxidation potential of 1.4 V (vs. RHE) was exerted on the CB electrodes in 0.5 mol·L^−1^ H_2_SO_4_ solution for different durations (0–200 s). Then, the anodized CB electrodes were rinsed with deionized water and dried at 60 °C for later use. Under certain cases hereafter, the anodized CB electrode is referred to as the CB (X) electrode, where X indicates the time of anodization.

### 2.3. Characterization and Testing Methods

Scanning electron microscopy (SEM, Hitachi s-4800) was used to characterize the surface morphology of the CB electrodes. The species and their composition on the surface of the CB electrodes were measured by X-ray photoelectron spectroscopy (XPS, k-alpha+). The contact angles of the CB electrodes were determined using an optical contact angle measuring instrument (OCA15EC).

Different CB electrodes were tested using an in-house cell, which has an anode chamber and cathode chamber separated by a Nafion 115 membrane, with a solution containing 3.48 mol·L^−1^ (NH_4_)_2_SO_4_ as the anolyte and a solution containing 0.41 mol·L^−1^ H_2_SO_4_ and 3.18 mol·L^−1^ (NH_4_)_2_SO_4_ as the catholyte to simulate an APS electrosynthesis environment. The working electrode (WE), counter electrode (CE), and reference electrode (RE) were the CB electrode, platinum sheet, and Ag/AgCl electrode, respectively. These electrodes were connected to an electrochemical workstation (PARSTAT4000) (see Figure S1 in Supplementary Materials). Oxygen or air was delivered to the CB electrode from beneath it in the form of bubbles generated via an aerator (Zhejiang Sensen Industry Co., Ltd., Zhejiang, China). In this experiment, we used a cathode with an area of 5 cm^2^ and an anode with an area of 1 cm^2^. When the cathode current density is 40–160 mA/cm^2^, the corresponding anode current density is 200–800 mA·cm^2^. In this condition, the ammonium persulfate could be synthesized with a high current efficiency. Chronopotentiometry curves were recorded at current densities ranging from 40 to 160 mA·cm^−2^. Linear sweep voltammetry (LSV) curves were also obtained with a scanning range of 1 to −0.4 V (vs. RHE) at a speed of 10 mV·s^−1^. The setup used was the same as that used in chronopotentiometry, except that the Pt sheet was replaced by a piece of carbon paper as the counter electrode, because the Pt electrode would be dissolved if it were used as an anode in electrolytes [33], and there is a risk that the dissolved Pt ion could migrate to the cathode and be reduced on the cathode.

APS electrosynthesis was carried out using the same setup at 30 °C, with the exceptions that a smaller platinum sheet (1 × 1 cm^2^) was used as the anode and 0.7 g·L^−1^ ammonium polyphosphate was added to the anolyte as an oxygen evolution inhibitor to increase the Faraday efficiency of the APS production. The anolyte was sampled at regular intervals and the APS concentration was determined via titration, as in ref. [32].

## 3. Results and Discussion

**Figure 1:**
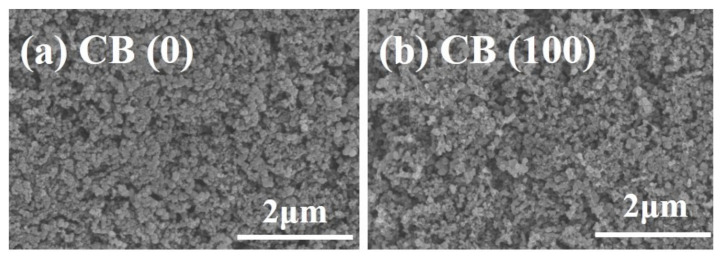
SEM images of the CB electrodes before (**a**) and after (**b**) in situ anodization. The oxidation potential was 1.4 V.

The CB electrodes have a porous structure, which is desirable for either HER or ORR. In addition, it can be seen that the surface morphology of the electrodes had hardly any noticeable changes after the mild anodization at 1.4 V (see Figure 1a,b). However, the chemical composition on the surface of the CB electrodes was changed by the anodization, as revealed by XPS.

**Figure 2:**
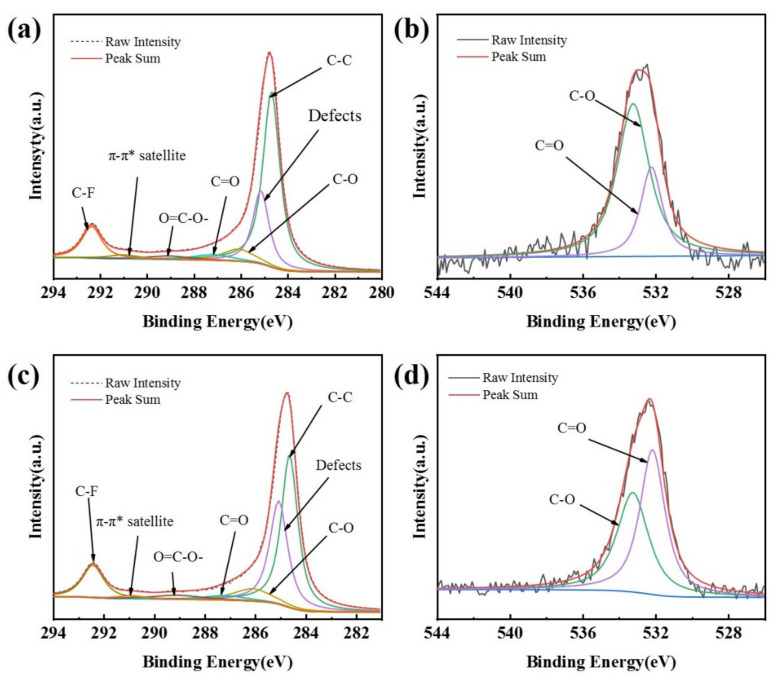
XPS spectra of C1s for CB (0) (**a**), C1s for CB (100) (**c**), O1s for CB (0) (**b**), and O1s for CB (100) (**d**).

The peaks of both C1s and O1s, at 285.5 eV and 532.5 eV, respectively, appear in the XPS spectra of the CB (0) electrode and the CB (100) electrode (Figure S2). A larger O1s peak of the CB (100) electrode over the CB (0) electrode can be noticed (Figure S2, insert), indicating an increase in oxygen-containing functional groups on the surface of carbon black in the CB (100) electrode as a result of anodization [34,35]. High-resolution XPS spectra reveal the specific oxygenated species on the CB (0) electrode and the CB (100) electrode, as shown in Figure 2. The C1s spectra can be deconvoluted into seven peaks [26,34,35,36,37,38], including C–C related to graphitic carbon at 284.8 eV, amorphous carbon defects on carbon black surface at 285.4 eV, C–O (286.4–286.9 eV), C=O (287.8 eV), O=C−OH (289.3 eV), the characteristic shakeup line of carbon (π−π* transition) in aromatic compounds at 291.2 eV, and C–F at 292.3 eV. The O1s spectra can be deconvoluted into two peaks [26,36], which are assigned to C=O (532.2 eV) and C–O (533.2 eV), respectively.

The data obtained from XPS measurement are summarized in Table 1, which shows that the oxygenated species on the surface of the CB electrodes increases with the time of anodization. As the anodization time increases from 0 s to 200 s, the oxygen content increases from 1.19% to 4.33% and the content of hydrophilic groups (C=O, O=C−OH) also increases. There are more oxygen-containing groups and defect sites on the surface of the CB (100) electrode as compared with the CB (0) electrode (Figure 2, Table 1). These oxygen-containing groups and defect sites could be easily adsorbed by O_2_ and could serve as active sites [26,36]. The XPS measurement shows that the electrochemical modification of the CB electrode adopted in this research is very effective at introducing oxygen-containing groups (C=O, O=C−OH) on the surface of carbon black, considering the short time needed to cause a noticeable increase in oxygenated species.

**Figure 3:**
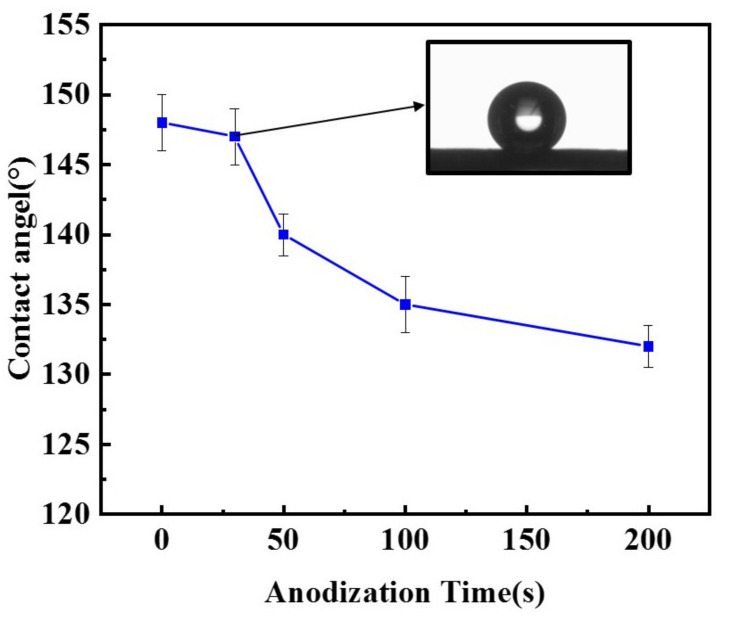
Contact angle of CB electrodes anodized at 1.4 V for different durations (0–200 s). Insert: An optical image of a single water drops on the surface of a CB electrode anodized for 30 s.

The increase in oxygenated species on the surface of carbon black, in turn, renders the CB electrodes less hydrophobic. The water contact angle of the surface of the CB electrodes anodized for different durations (0–200 s) is presented in Figure 3. It shows that the contact angle of the CB electrodes decreased with the increase in anodizing time. The contact angle falls from the initial angle of approximately 148° to about 132° after 200 s of anodization. The slightly increased hydrophilicity would facilitate the wetting of the electrode by the electrolyte and thus increase the electrochemical active area. However, even after the longest anodization time, the CB electrodes are still hydrophobic, with a contact angle much larger than 90°, largely because of the presence of dispersed PTFE, which is hardly affected by the anodization. This is important for the CB electrodes to be used in an ORR, as they should be able to catch the passing oxygen or air bubbles so as to make use of them.

**Figure 4:**
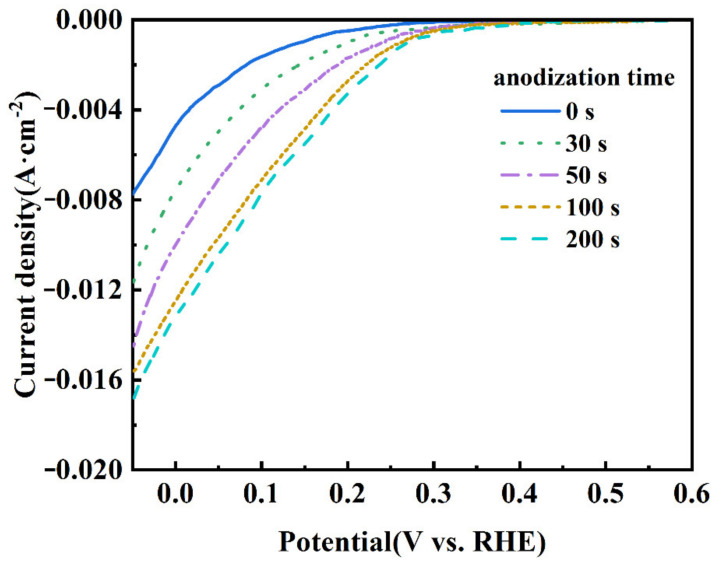
LSV curves of CB electrodes anodized for 0, 30, 50, 100, and 200 s at 1.4 V. The electrolyte was saturated with O_2_ and the scanning rate was 10 mV s^−1^.

Figure 4 shows the LSV curves of the CB electrodes anodized at 1.4 V for different durations. It is seen that the onset potential of the ORR shifts to the direction of positive potential. The ORR onset potential of the CB (0) electrode is about 0.27 V, while that of the CB (100) electrode positively shifts to 0.38 V. Moreover, the cathodic current increases with the anodization time. These results indicate that the ORR activity of the electrode is improved by the mild anodization [39]. The improvement is dramatic within the first 100 s but slows down afterwards, suggesting that the number of active sites for the ORR does not increase proportionally with the time of anodization.

In the process of the ORR, gaseous/dissolved oxygen was adsorbed at the surface of the catalyst. There are two typical adsorption modes for an oxygen molecule: the side-on mode (known as the Yeager model) and the end-on mode (known as the Pauling model). The mode of oxygen adsorption has a great influence on the reduction pathway; the end-on adsorption of oxygen is easier to pass through the two-electron reduction path, while the side-on adsorption is favorable to the four electron reduction path of oxygen [40,41]. The electropositive carbon atom in carbonyl (C=O) or carboxyl (O=C−OH) groups could preferentially adsorb the oxygen molecule in the end-on mode, which is beneficial for catalyzing the ORR in a two-electron pathway [42]. The LSV results show that the CB (100) electrode exhibits a higher ORR activity compared with the CB (0) electrode, which can be attributed to its having more C=O and O=C−OH.

**Figure 5:**
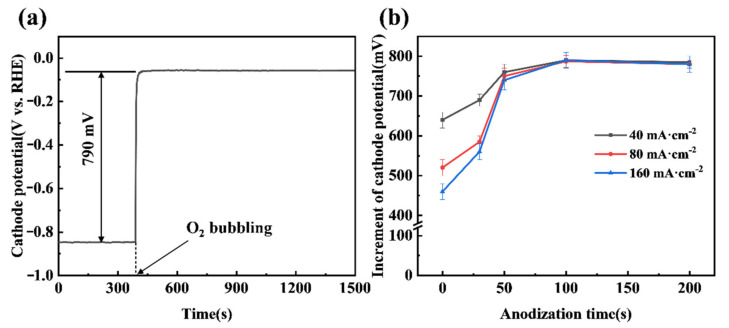
(**a**) The chronopotentiometry curve of the CB (100) electrode at a cathode current density of 40 mA·cm^−2^; (**b**) Effect of anodization time on the chronopotentiometry curve of CB electrodes at different current densities. The O_2_ flow rate was 200 mL·min^−1^.

A significant increase in cathode potential can be achieved when the anodized CB electrode shifts from a HER mode to an ORR mode, as shown in Figure 5a. The chronopotentiometry curve demonstrates that bubbling O_2_ gas to the CB (100) electrode can lead to the electrode potential jumping from −0.85 V to −0.06 V, an increment of 790 mV, at a constant current density of 40 mA cm^−2^. It is also noted that the potential of the CB (100) electrode in the ORR mode can be very stable, indicating the ability of the anodized CB electrode to effectively trap O_2_ gas bubbles and facilitate the ORR. The duration of anodization of the CB electrode has an impact on the increment of potential when shifting from the HER mode to the ORR mode, as shown in Figure 5b. While the increment approaches a steady 790 mV after 100 s of anodization, less than 50 s of anodization results in noticeable lessened cathode potential increments. Presumably, an insufficiently anodized CB electrode lacks sufficient ORR active sites, meaning that the shift from the HER mode to the ORR mode cannot be completed and the two simultaneous reactions result in a mixed potential. This might explain why the lessened cathode potential increments become more severe with the increase in current passing the electrode (see Figure 5b). Practically, the results from Figure 5b also suggest that around 100 s is the best duration of anodization in terms of achieving the highest cathode potential increment.

**Figure 6:**
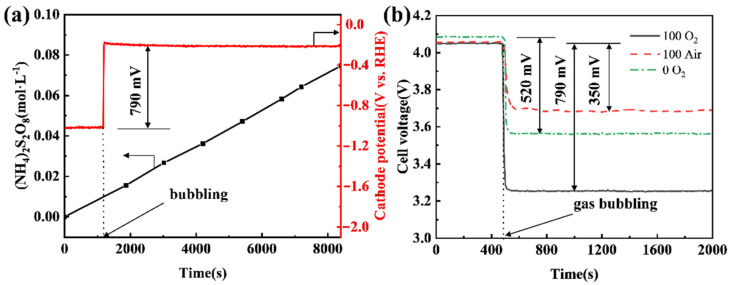
(**a**) Chronopotentiometry curve of the CB (100) electrode and the concentration of APS in anode solution; (**b**) effect of air or O_2_ on cell voltage when using CB (0) or CB (100) as the cathode. The anode current density was 400 mA·cm^−2^, and the cathode current density was 80 mA·cm^−2^, oxygen and air flow rates were all 200 mL·min^−1^, and the distance between the anode and cathode was 2.0 cm.

To evaluate the energy-saving potential of shifting a cathode reaction from a HER mode to an ORR mode in a practical electrooxidation process, APS electrosynthesis was carried out using different CB electrodes as a cathode under different conditions. It can be seen that the APS concentration increases linearly with the time of electrolysis, while the potential of the CB (100) electrode increases by 790 mV when shifting from the HER mode to the ORR mode by bubbling pure oxygen (Figure 6a). This translates into a cell voltage decrease from 4.05 V to 3.26 V (see Figure 6b). If air replaces pure oxygen, the potential increment of the CB (100) cathode falls to 350 mV (Figure 6b). This is understandable, as noticeably increased overpotential would be expected to drive the ORR at the same current when the concentration of oxygen drops around 80%. If the CB (0) electrode, i.e., a pristine CB electrode that has not been anodized, is used as the cathode in APS electrosynthesis, shifting from the HER mode to the ORR mode by bubbling pure oxygen leads to a cathode potential increment of 520 mV, or a fall of cell voltage from 4.09 V to 3.57 V. It is clear from the data shown in Figure 6b that the anodization of a CB electrode is very effective in improving its ORR performance, but much less so for its HER performance.

Compared with using a CB (0) electrode under the HER mode, the use of a CB (100) electrode under the ORR mode with O_2_ bubbling leads to the cell voltage of APS electrosynthesis reducing from 4.09 V to 3.26 V (Figure 6b), which means a 20.3% saving in electricity. Even if air is bubbled under the ORR mode, a 9.0% saving in electricity can be achieved. Given that the Faraday efficiency of APS production in the test was 63.1% (calculated using the data in Figure 6a), 323 kWh per tonne of APS would be saved if the ORR mode with O_2_ bubbling were adopted and 143 kWh per tonne of APS would be saved if there were air bubbling. Moreover, Lavrenko et al. [43] used a silver gas-diffusion cathode to catalyze the ORR in the electrosynthesis of ammonium persulfates and a reduced cell voltage of 0.7 V was achieved. In our work, the cell voltage can be reduced by 0.79 V using the cheap CB cathode, and there is no leakage of electrolyte from the cathode.

## 4. Conclusions

To lower the energy consumption of electrochemical oxidation processes that accompany hydrogen evolution, a simple yet effective method is demonstrated to enable the cathode to shift from the original HER mode into an ORR mode. To work in an ORR mode, the mildly anodized CB electrode catches and reduces oxygen supplied via bubbling pure oxygen or air from beneath it. As such, there is no need to build new electrolysis cells and no possible leakage of electrolytes from the O_2_ gas electrode. The viability of this method is exemplified by the electrosynthesis of APS, in which a 20.3% saving in electricity can be achieved upon shifting from the original HER mode to an ORR mode. Considering its simplicity and effectiveness, this method is very promising in a wide variety of applications.

## Figures and Tables

**Table 1:** The contents of O and oxygen-containing functional groups on the surface of different CB electrodes.

Electrodes	O Atomic Percent (%)	Oxygen-Containing Functional Groups Atomic Percent (%)	
		**C=O**	**C–O**
CB (0)	1.19	0.34	0.85
CB (50)	1.76	0.79	0.97
CB (100)	2.48	1.34	1.14
CB (200)	4.33	3.03	1.30

## Data Availability

Not applicable.

## Supplementary Materials

The following are available online at https://www.mdpi.com/article/10.3390/ma14112841/s1, Figure S1: Schematic diagram of the device for measuring cathode potential; Figure S2: XPS general spectra and O1s spectrum of CB (0) and CB (100) electrodes (inset).
