# Supplementary material for: A Facile Method to Realize Oxygen Reduction at the Hydrogen Evolution Cathode of an Electrolytic Cell for Energy-Efficient Electrooxidation
Source: Materials (Basel). 2021 May 26;14(11):2841. doi: 10.3390/ma14112841 (PMC8198103; doi:10.3390/ma14112841)
Supplement: Supplementary file 1 [file materials-14-02841-s001.zip › materials-1216188-supplementary.pdf]

materials MDPI

*Supporting information*

# A Facile Method to Realize Oxygen Reduction at the Hydrogen Evolution Cathode of an Electrolytic Cell for Energy-Efficient Electrooxidation

**Zhiqiang Zhao, Lu Liu, Luofu Min, Wen Zhang and Yuxin Wang ***

State Key Laboratory of Chemical Engineering, Tianjin Key Laboratory of Membrane Science and Desalination Technology, School of Chemical Engineering and Technology, Tianjin University, Tianjin 300072, China; zqzhao@tju.edu.cn (Z.Z.); 1016207153@tju.edu.cn (L.L.); minluofu@tju.edu.cn (L.M.); zhang_wen@tju.edu.cn (W.Z.)

* Correspondence: yxwang@tju.edu.cn

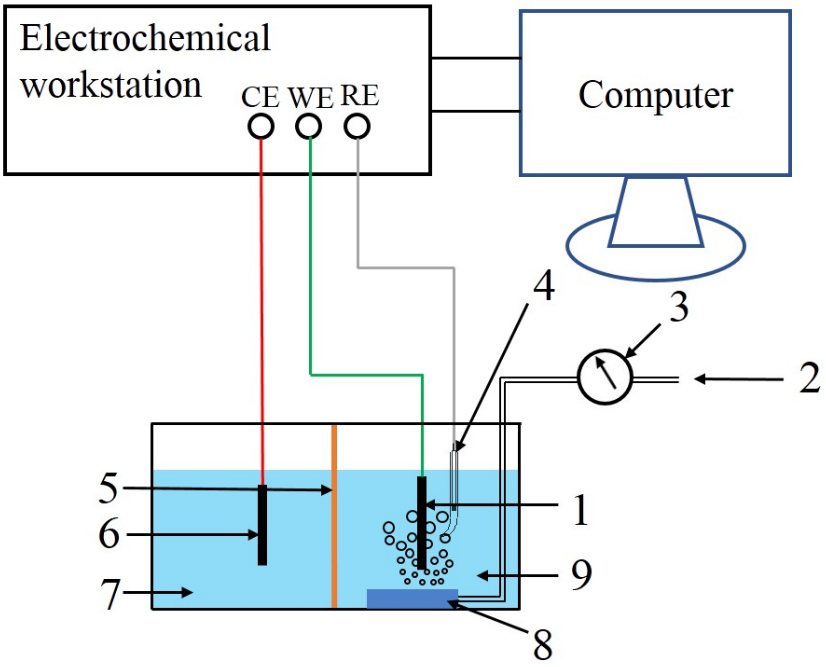


**Figure S1.** Schematic diagram of the device for measuring cathode potential. 1-WE, 2-gas inlet, 3-rotameter, 4-RE, 5-separator, 6-CE, 7-anolyte, 8-aerator, 9-catholyte.

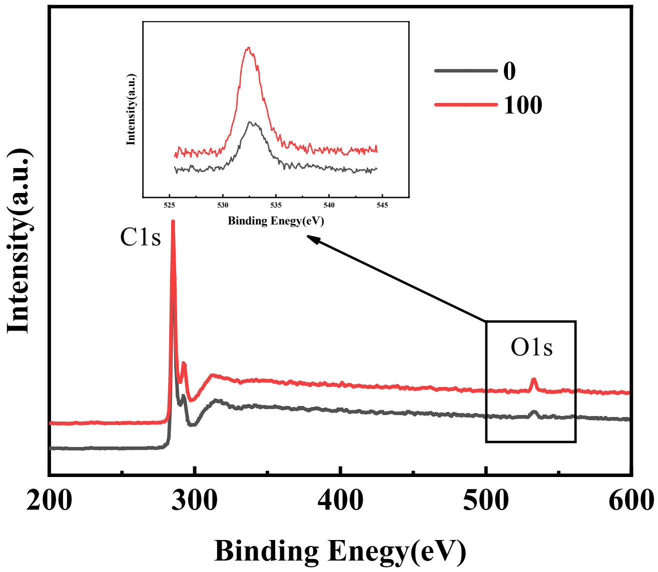


**Figure S2.** XPS general spectra and O1s spectrum of CB (0) and CB (100) electrodes (inset).
